# Zoonotic Filariasis Caused by Novel *Brugia* sp. Nematode, United States, 2011

**DOI:** 10.3201/eid2007.131654

**Published:** 2014-07

**Authors:** Alberto Enrique Paniz-Mondolfi, Teresa Gárate, Christine Stavropoulos, Wen Fan, Luis Miguel González, Mark Eberhard, Fred Kimmelstiel, Emilia Mia Sordillo

**Affiliations:** Yale University School of Medicine, New Haven, Connecticut, USA (A.E. Paniz- Mondolfi);; St. Luke’s-Roosevelt Hospital Center of Columbia University College of Physicians and Surgeons, New York, New York, USA (A.E. Paniz-Mondolfi, C. Stavropoulos, W. Fan, F. Kimmelstiel, E.M. Sordillo);; Servicio Autonomo Instituto de Biomedicina/Instituto Venezolano de los Seguros Sociales, Caracas, Venezuela (A. Paniz Mondolfi);; Instituto de Salud Carlos III, Madrid, Spain (T. Gárate, L.M. González);; Centers for Disease Control and Prevention, Atlanta, Georgia, USA (M. Eberhard)

**Keywords:** Brugia sp. Nov, zoonotic filariasis, North America, New York, United States, parasites, nematode

**To the Editor:** Zoonotic brugian filariasis is an incidental infection of humans with *Brugia* spp. nematodes that primarily parasitize nonhuman vertebrates, rarely humans (*1*–*3*). In contrast to classical lymphatic filariasis caused by *B. malayi* and *B. timori*, which are found in Asia, most zoonotic *Brugia* infections have been reported from the northeastern United States (*2*,*3*) or South America (*3*). We report a case of symptomatic brugian infection in a New York City resident who had not traveled to the Eastern Hemisphere.

In 2011, a 53-year-old White man first noted tenderness and swelling behind his penis and in his right groin after having fallen 3 months earlier. The tenderness was relieved by nonsteroidal antiinflammatory drugs, but the swelling continued; an oral antimicrobial drug, prescribed for presumed cellulitis, produced no improvement. At the time of examination, the patient had no fever or other signs or symptoms. Only a 3.0-cm × 3.0-cm firm, nonfixed right inguinal nodule without warmth or tenderness was noted. Laboratory findings were remarkable for total leukocytes of 6.4 × 10^9^, eosinophilia (12%, 600 cells/mm^3^), decreased hemoglobin level (10.0 g/dL), and low hematocrit of 31.2%. An excisional biopsy sample revealed intralymphatic adult nematodes with viable-appearing microfilaria (Technical Appendix Figure).

The patient had been born and raised in Champlain, Illinois, and had resided in the Bronx, New York, since 1979; he had no history of travel to filariasis-endemic regions. Characteristics of the adult worms and microfilaria were most consistent with those of *Brugia* spp., which was surprising because classical brugian lymphatic filariasis seems to be limited to Asia (*B. malayi*) and Indonesia (*B. timori*) (*4*,*5*). However, the adult filariae were smaller than expected for *B. malayi* or *B. timori* nematodes, prompting consideration of zoonotic filariasis (*1*,*6*). The adult worms and microfilaria seemed to be viable, although zoonotic *Brugia* spp. in histologic sections often appear degenerated (*1*,*2*,*6*). The diameters of the adult worms were similar to those reported from South America (females 90–100 μm, males 50 μm) (*7*,*8*) rather than those from North America (females 35–75 μm, males 32–52 μm) (*1*). Peripheral blood was repeatedly negative for microfilaria. Serum sent to the Centers for Disease Control and Prevention (Atlanta, GA, USA) for ELISA testing for *B. malayi* anti-filarial IgG 4 showed optical density of 0.13, below the ELISA cutoff for filariasis.

Because micromorphologic information was not adequate for species identification, paraffin-embedded biopsy specimens were submitted for molecular testing. Genomic DNA extracted from paraffin-embedded tissue with the QIAamp DNA–formalin-fixed, paraffin-embedded tissue procedure was amplified by using the primer sets DiBu-F(5′-GCTAGATATGCTACCAACAAAA-3′)/ITS1 R(5′-CTCAATGCGTCTGCAATTCGC-3′) and BuF2-(5-CATTTATGCTAGATATGCTACCAAC-3′)/ITS1-R. The products were fractionated on 2% agarose gel and stained with ethidium bromide. The internal transcribed spacer (ITS) 1 PCR product (182 bp) was automatically sequenced by using the same primers used for PCR. Lasergene software (DNASTAR, Madison, WI, USA) was used to align the sequences obtained with *Brugia* spp. sequences deposited in GenBank; detailed sequence comparison identified the isolate as a novel *Brugia* (Nematoda: Onchocercidae) species closely related to *B. pahangi* and *B. malayi* (Figure). The ITS-1 sequence was submitted to the EMBL Nucleotide Sequence Database (accession no. HE856316).

**Figure:**
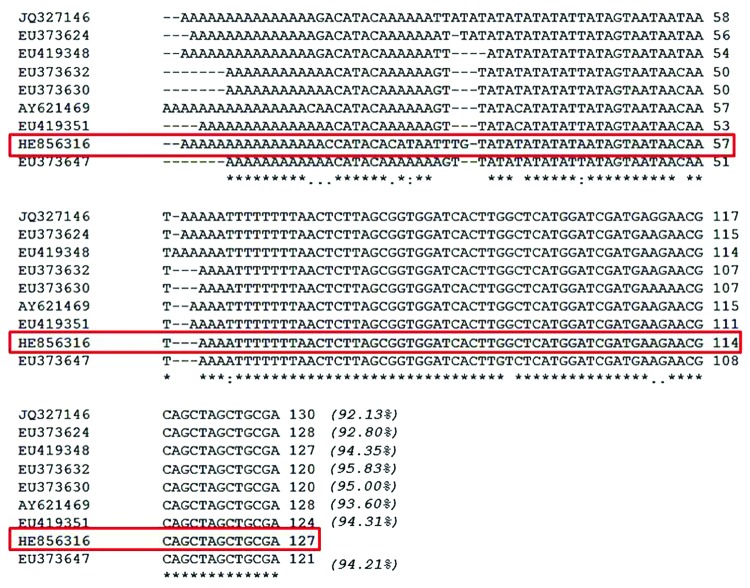
Pile-up of partial ribosomal DNA sequences from *Brugia* NY strain (HE856316) and from other related *Brugia* spp. strains and clones, *B. malayi* BM28 (JQ327146), *B. malayi* C27Cat5 (EU373624), *B. pahangi* C61CAT5 (EU419348), *B. pahangi* C14Cat6 (EU373632), *B. pahangi* C7Cat6 (EU373630), *B. pahangi* Bp-1 (AY621469), *B. pahangi* C46CAT5 (EU419351), and *B. pahangi* C27Cat7 (EU373647). The *Brugia* NY strain (HE856316) is enclosed in a red box; asterisks (*) indicate conserved residues; periods (.) indicate nucleotide changes; colons (:) indicate nucleotide changes just in the *Brugia* NY isolate; hyphens (-) are included in the sequences to maximize the comparisons among the 9 DNA molecules. Italicized numbers in parentheses indicate the percentage of similarity with the *Brugia* NY isolate.

Removal of an affected lymph node without additional treatment is often considered sufficient treatment for zoonotic filariases. However, for the patient reported here, persistence of inguinal swelling prompted a repeat biopsy 4 months later; the specimen again demonstrated reactive follicular hyperplasia, although no parasites were seen. Because the patient’s initial clinical signs and subsequent persistent adenopathy were reminiscent of unilateral lymphadenitis, lymphangitis, and induration that are typical of *B. malayi* or *B. timori* filariasis, and the microfilariae in the original biopsy sample appeared to be viable, we empiricially prescribed a standard dosage of oral doxycycline for 6 weeks, followed by single doses of ivermectin at 400 μg/kg and 800 mg albendazole. The patient has been well, without further adenopathy or eosinophilia, for >2 years. Because adult filariae can live for >10 years, the place of acquisition cannot be stated with certainty.

The prevalence of zoonotic infection with *Brugia* spp. nematodes is unknown. Many reported cases are asymptomatic or diagnosed incidentally during evaluation for persistent adenopathy (*1*–*3*). Conversely, differentiation of zoonotic from classical filariasis is unlikely in disease-endemic areas; most cases published since the initial 1962 case report (*1*) occurred in the United States. Most case-patients were from the Northeast, including New York (8 cases), Massachusetts, Pennsylvania, Connecticut, and Rhode Island (3 cases each) (*1*,*2*); single cases have been identified in Michigan, Ohio, North Carolina, Oklahoma, New Jersey, Louisiana, Florida, and California (*1*,*2*). Four other cases have been reported: 3 in South America (Colombia, Brazil, Peru) (*3*,*7*,*8*) and 1 in Africa (Ethiopia) (*9*). Only a few *Brugia* species have been identified, including *B. leporis*, found in rabbits in the northeastern United States (*1*,*10*); *B. beaveri*, found in raccoons and bobcats in the southern United States; and *B. guyanensis*, found in coatimundi and other vertebrates in South America (*8*). Definitive identification with molecular techniques will better identify causative species and help clarify many of the ecologic and epidemiologic questions surrounding zoonotic filarial infections.

Technical Appendix Histocytologic appearance of *Brugia* nematodes in 53-year-old White man from New York, USA.

## References

[1] Orihel TC, Eberhard ML. Zoonotic filariasis. Clin Microbiol Rev. 1998;11:366–81 . 9564568 10.1128/cmr.11.2.366 PMC106837

[2] Eberhard ML, DeMeester LJ, Martin BW, Lammie PJ. Zoonotic *Brugia* infection in western Michigan. Am J Surg Pathol. 1993;17:1058–61 . 10.1097/00000478-199310000-00012 8372943

[3] Orihel TC, Beaver PC. Zoonotic *Brugia* infections in North and South America. Am J Trop Med Hyg. 1989;40:638–47 . 2662786 10.4269/ajtmh.1989.40.638

[4] Taylor MJ, Hoerauf A, Bockarie M. Lymphatic filariasis and onchocerciasis. Lancet. 2010;376:1175–85. 10.1016/S0140-6736(10)60586-7 20739055

[5] Schneider MC, Aguilera XP, Barbosa da Silva Junior J, Ault SK, Najera P, Martinez J, Elimination of neglected diseases in Latin America and the Caribbean: a mapping of selected diseases. PLoS Negl Trop Dis. 2011;5:e964. 10.1371/journal.pntd.0000964 21358810 PMC3039687

[6] Gutierrez Y. Diagnostic features of zoonotic filariae in tissue sections. Hum Pathol. 1984;15:514–25. 10.1016/S0046-8177(84)80004-0 6539296

[7] Kozek WJ, Reyes MA, Ehrman J, Garrido F, Nieto M. Enzootic *Brugia* infection in a two-year old Colombian girl. Am J Trop Med Hyg. 1984;33:65–9 . 6696186 10.4269/ajtmh.1984.33.65

[8] Baird JK, Neafie RC. South American brugian filariasis: report of a human infection acquired in Peru. Am J Trop Med Hyg. 1988;39:185–8 . 3407837 10.4269/ajtmh.1988.39.185

[9] Menéndez MC, Bouza M. *Brugia* species in a man from western Ethiopia. Am J Trop Med Hyg. 1988;39:189–90 . 3407838 10.4269/ajtmh.1988.39.189

[10] Beaver PC, Orihel TC. Human infection with filariae of animals in the United States. Am J Trop Med Hyg. 1965;14:1010–29 . 5891453 10.4269/ajtmh.1965.14.1010

