# Supplementary material for: Zoonotic Filariasis Caused by Novel Brugia sp. Nematode, United States, 2011
Source: Emerg Infect Dis. 2014 Jul;20(7):1248–50. doi: 10.3201/eid2007.131654 (PMC4073870; doi:10.3201/eid2007.131654)
Supplement: Technical Appendix — Histocytologic appearance of Brugia nematodes in 53-year-old White man from New York, USA. [file 13-1654-Techapp-s1.pdf]

Article DOI: http://dx.doi.org/10.3201/eid2007.131654

Zoonotic Filariasis Caused by Novel *Brugia* sp. Nematode, United States

Technical Appendix

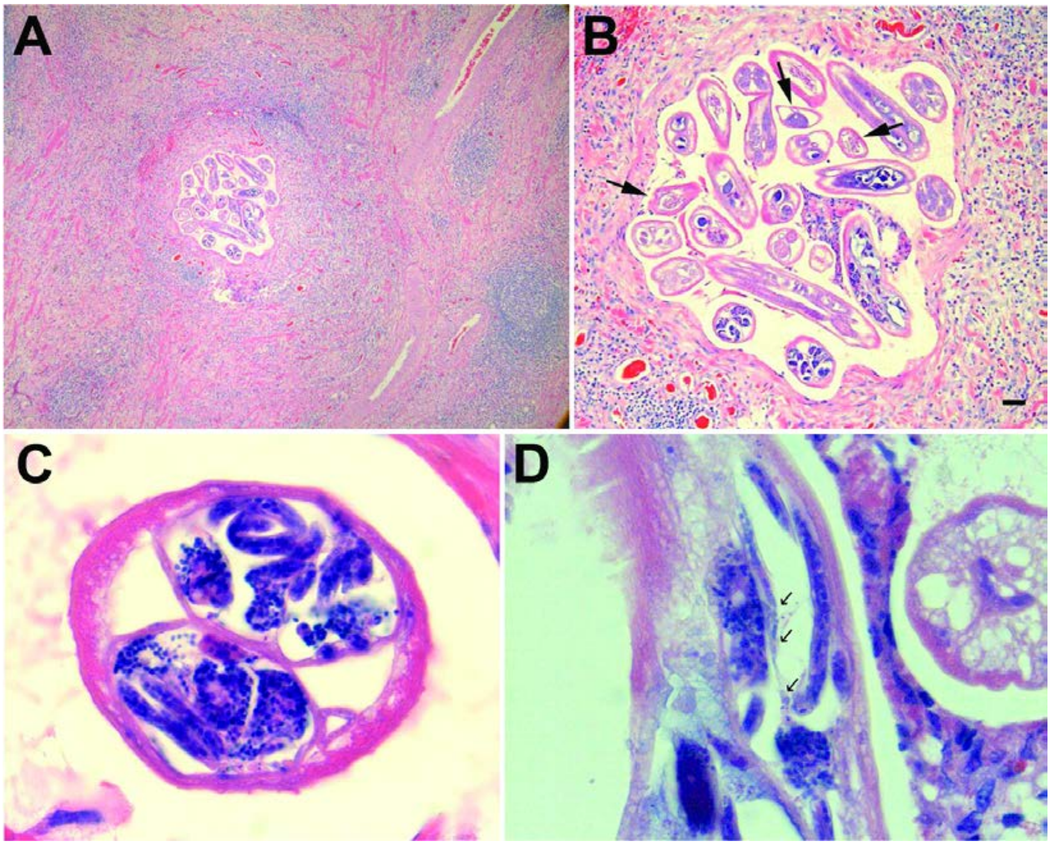

Figure. Histocytologic appearance of *Brugia* nematodes in 53-year-old White man from New York, USA. A) Male and female nematodes within the lymph node with reactive follicular hyperplasia and a lymphohistiocytic granulomatous infiltrate permeated by few scattered eosinophils. Hematoxylin and eosin stain, magnification ×5. B) Male (arrows) and female worms in varying longitudinal and transverse plains. Note that males are smaller than females and have a pseudocoelomic cavity containing a single reproductive tube and intestine. Females are larger and contain paired uterine tubes. Scale bar = 50 µm, hematoxylin and eosin stain, magnification ×20. C) Gravid female. The pseudocoelom is mostly occupied by 2 uterine tubes filled with developing eggs and microfilariae, and the small simple intestine. Note the thin cuticle, which becomes thickened and more prominent over the lateral cords; the low musculature consists of few muscle cells. Hematoxylin and eosin stain, magnification ×40. D) Mature microfilariae in the uterus. The arrows point out the 3 posterior nuclei. Note that the width of the microfilariae ranged from 4.5 µm to 5.5 µm. Hematoxylin and eosin stain, magnification ×100.
